# Regional Stiffness and Hardening Indices: New Indicators Derived from Multidimensional Dynamic CTA for Aneurysm Risk Assessment

**DOI:** 10.1002/advs.202400653

**Published:** 2024-10-25

**Authors:** Tianming Huang, Xiaoyu Qi, Lan Cao, Ming Yang, Huan Luo, Qin Li, Peidong Qian, Jia Lu, Ziqiao Lei, Yuanming Luo, Chao Yang

**Affiliations:** ^1^ Department of Technology Boea Wisdom (Hangzhou) Network Technology Co., Ltd. Hangzhou 310000 China; ^2^ Department of Vascular Surgery Union Hospital Huazhong University of Science and Technology Wuhan 43022 China; ^3^ Department of Radiology Hubei Province Key Laboratory of Molecular Imaging Union Hospital Huazhong University of Science and Technology Wuhan 43022 China; ^4^ Department of Mechanical Engineering The University of Iowa Iowa City 52242 USA

**Keywords:** aneurysm, biomechanical analysis, in vivo, logistic regression, MD CTA

## Abstract

Two indices, indicating the regional average stiffness and the pace of strain hardening respectively, are derived from the nonlinear stress–strain behavior obtained from biomechanical analysis of aneurysm. A comprehensive method based on electrocardiographic‐gated multidimensional dynamic computed tomography angiography (MD CTA) is developed for extracting these mechanical characteristics in vivo. The proposed indices are evaluated by 26 cases including 9 healthy, one aortosclerosis, and 16 abdominal aortic aneurysm cases. The difference of *SSI* and *dSSI* value between aneurysmal and healthy groups is up to orders in magnitude. Significant correlation of these indices with the clinical indicator of aneurysm diameter is found. Logistic models based on these indices are capable to sharply discriminate the healthy and the aneurysmal arteries with AUC>0.98. This work introduces new tools and new indices for aortic mechanical assessment which may shed light on understanding the mechanical condition, pathological state and eventually benefit clinical decision‐making.

## Graphical abstract

Two new indicators are derived from nonlinear stress–strain behavior obtained through in vivo biomechanical analysis of patient‐specific multidimensional dynamic computed tomography angiography. The regional stiffness index indicates the average stiffness while the hardening index reflects the pace of strain hardening. Both indices sharply differentiate healthy and aneurysmal arteries, demonstrating their promise to guide aneurysm diagnosis and personalized treatment decisions.

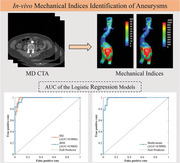

## 1 Introduction

Aneurysms, a critical medical condition characterized by the weakening and bulging of blood vessel walls, pose a significant risk of rupture and potentially fatal internal bleeding.^[^ ^1^, ^2^, ^3^, ^4^, ^5^, ^6^, ^7^, ^8^, ^9^ ^]^ Over the years, there has been ongoing research regarding the implication and significance of aortic wall mechanical conditions and tissue properties in evaluating the risk of aortic aneurysm rupture.^[^ ^2^, ^10^, ^11^, ^12^, ^13^, ^14^, ^15^, ^16^, ^17^ ^]^ These studies offer insights into the structural integrity, functional capacity, and pathological changes that occur during the formation and progression of aneurysms.^[^ ^18^, ^19^, ^20^, ^21^, ^22^ ^]^ Currently, measurement of aneurysm mechanical properties predominantly relies on in vitro tissue testing. Such invasive approaches clearly cannot be utilized for clinical patient‐specific assessments.

Computed tomography angiography (CTA) has been a widely used imaging technique for visualizing aneurysms, providing fast and high‐resolution images of the lumen, or the inner space of the vessel.^[^ ^23^, ^24^ ^]^ However, a limitation of CTA is its inability to capture the thickness and structure of the vessel wall, which are crucial for determining the mechanical state of aneurysm wall. In addition, the presence of intraluminal thrombus (ILT), as well as its structure and history, would also greatly influence its properties.^[^ ^25^, ^26^, ^27^, ^28^, ^29^ ^]^ Most existing studies on the stress analysis of aneurysms based on CTA images rely on either the lumen geometry or assumed thickness/ILT, rather than the actual wall geometry.^[^ ^2^, ^4^, ^29^, ^30^, ^31^ ^]^

In response to this challenge, this study proposes a novel approach to estimate the mechanical characteristics of aneurysms using electrocardiographic‐gated multidimensional dynamic computed tomography angiography (MD CTA) images. MD CTA allows to capture the change in lumen geometry during cardiac cycles, which is used to compute the strain at the lumen boundary (referred to as the lumen strain hereafter). As there is no wall geometry data to support 3D stress analysis, we use a surrogate model that treats the surrounding material (wall/thrombus) as an elastic membrane. The “membrane tension” can be reliably predicted using inverse stress analysis.^[^ ^33^, ^34^, ^35^, ^36^, ^37^, ^38^ ^]^ The “membrane tension” is not the real stress in the tissue, and not directly used to assess the mechanical condition. Instead, we focus on the change of it over the deformation process, which is speculated to reflect the stiffness of the AAA structure. Various studies have indicated that aneurysm tissues not only show a higher stiffness but also a faster pace of strain hardening.^[^ ^10^, ^14^, ^16^, ^39^ ^]^ Here, we introduced two indices, a scaled stiffness index (*SSI*) and its hardening index, *dSSI*. The former reflects the regional stiffness of the AAA and the latter reflects the strain hardening pace. The hypothesis underlying this approach is that these surrogate measures can better differentiate aneurysms from healthy aorta.

**Figure 1:**
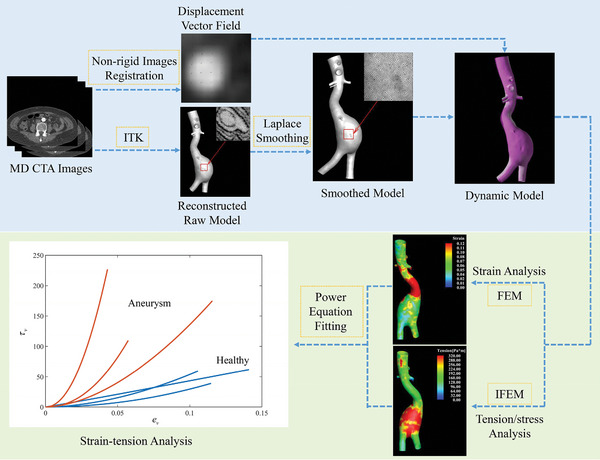
Flowchart of the biomechanical analysis.

To validate this hypothesis, we investigated 26 MD CTA scans encompassing cases of healthy abdominal aortas and AAAs in different sizes. **Figure** **1** illustrates the workflow. The surrogate properties derived from the MD CTA images are then compared with the current clinical aneurysm assessment, i.e., diameter. The main objective of this study is to demonstrate the feasibility and potential of using these properties as non‐invasive indicators of aneurysm mechanical behavior, advancing the understanding of aneurysm biomechanics and potentially improving clinical monitoring of aneurysm development based on routine CTA imaging data.

## 2 Results and Discussion

### 2.1 Registration Results

**Figure 2:**
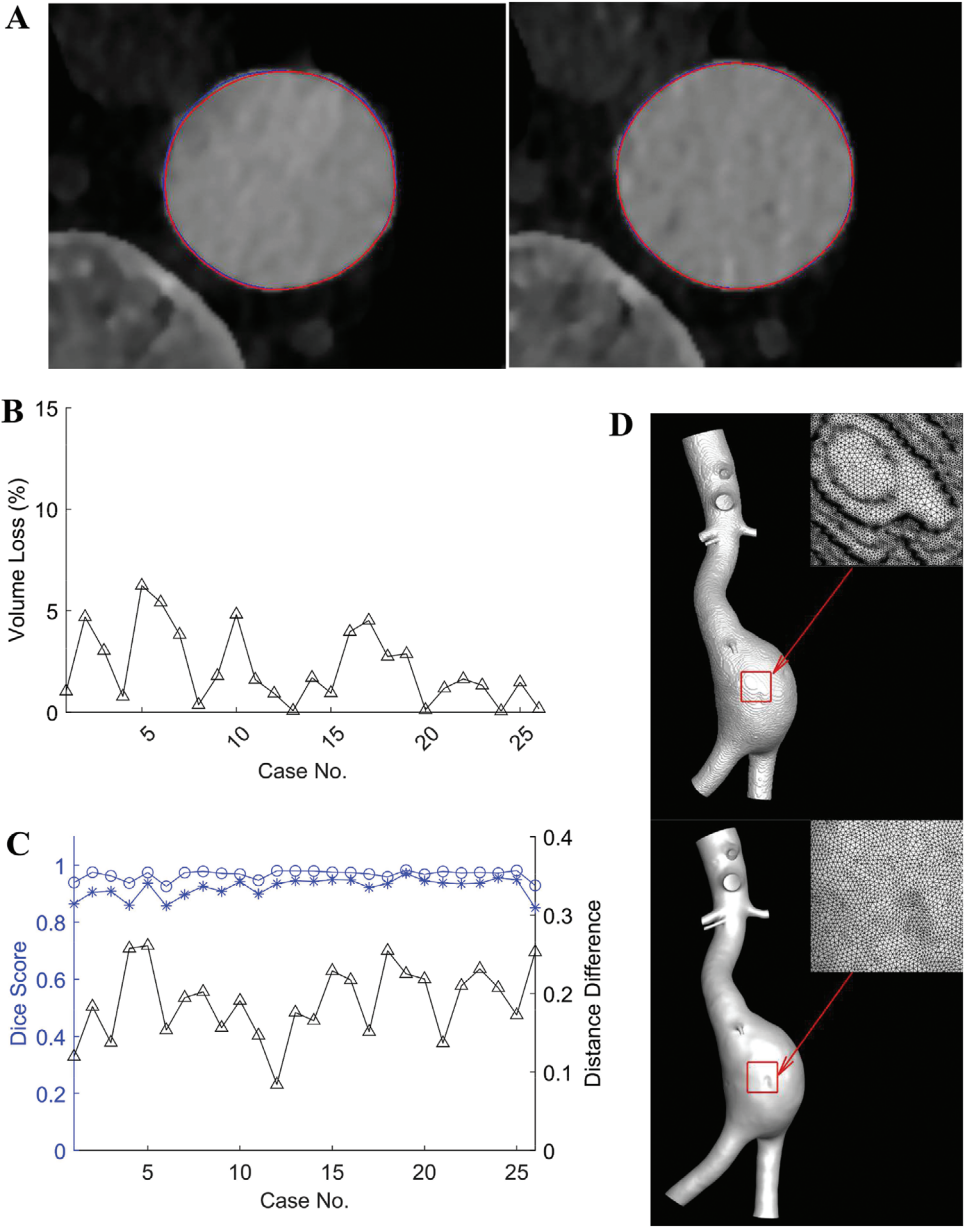
Results of registration, smoothing, and model reconstruction. A) Contours of a cross‐section in case No. 1, Blue line: manual labeling results; Red line: registration results of deformed mesh; Left: beginning phase of systole; Right: peak phase of systole. B) Volume loss caused by smoothing. C) Dice scores before and after registration, and mean distance difference between registration and manual annotation results at peak systole phase: “*” Dice score before registration; “o” Dice score after registration; “△” mean difference. D) STL models of case No. 12 before (upper) and after smoothing (lower).

MD CTA imaging enables obtaining dynamic aortic geometries over cardiac cycles. Starting from a selected reference configuration (the reference was generally set to the configuration at the first phase in an R‐R interval in this work), we used image registration to derive displacement fields with respect to the reference. This process is applied to all the dilating phases, which is detailed in Section 4. The displacements of each node are applied to the reference mesh to obtain a deforming mesh that corresponds over all dilating phases. **Figure** **2**A presents an example (case No. 1) of the aortic lumen wall at the beginning and peak phases, obtained from registration (blue) and manual annotation (red, the ground truth). The contours are in good agreement. The registration performance was evaluated by the Dice score, which is given in Figure 2C. All the registration results are in good quality with Dice score >0.92. The mean distance difference between registration results and manual annotation results is also presented in Figure 2C. For image based methods, the resolution is restricted by the voxel size which in our study is 0.3‐0.4 mm. The mean distance difference in all cases is less than a voxel dimension, and hence within the resolution limit.

### 2.2 Aortic Lumen Wall Reconstruction

3D surface geometry models of the aortic lumen wall were first described by the standard triangle language (STL) models. The initial models reconstructed directly from CTA images often contained numerous singular points on the surface. Hence, mesh smoothing process for the models was necessary. However, it is important to note that the smoothing process may cause structure shrinkage, which can result in a reduction of the model's volume compared to the original. To assess the impact of Laplace smoothing on the models, volume loss of all 26 cases was analyzed and given in Figure 2B. An example is given in Figure 2D to showcase the STL models before and after the Laplace smoothing (the remaining models are shown in Table S1, Supporting Information). The maximum volume loss is <6% in all cases. It could be found, as exampled in Figure 2D, that the smoothness of the STL model is significantly improved after smoothing.

### 2.3 Lumen Strain and Tension

**Figure 3:**
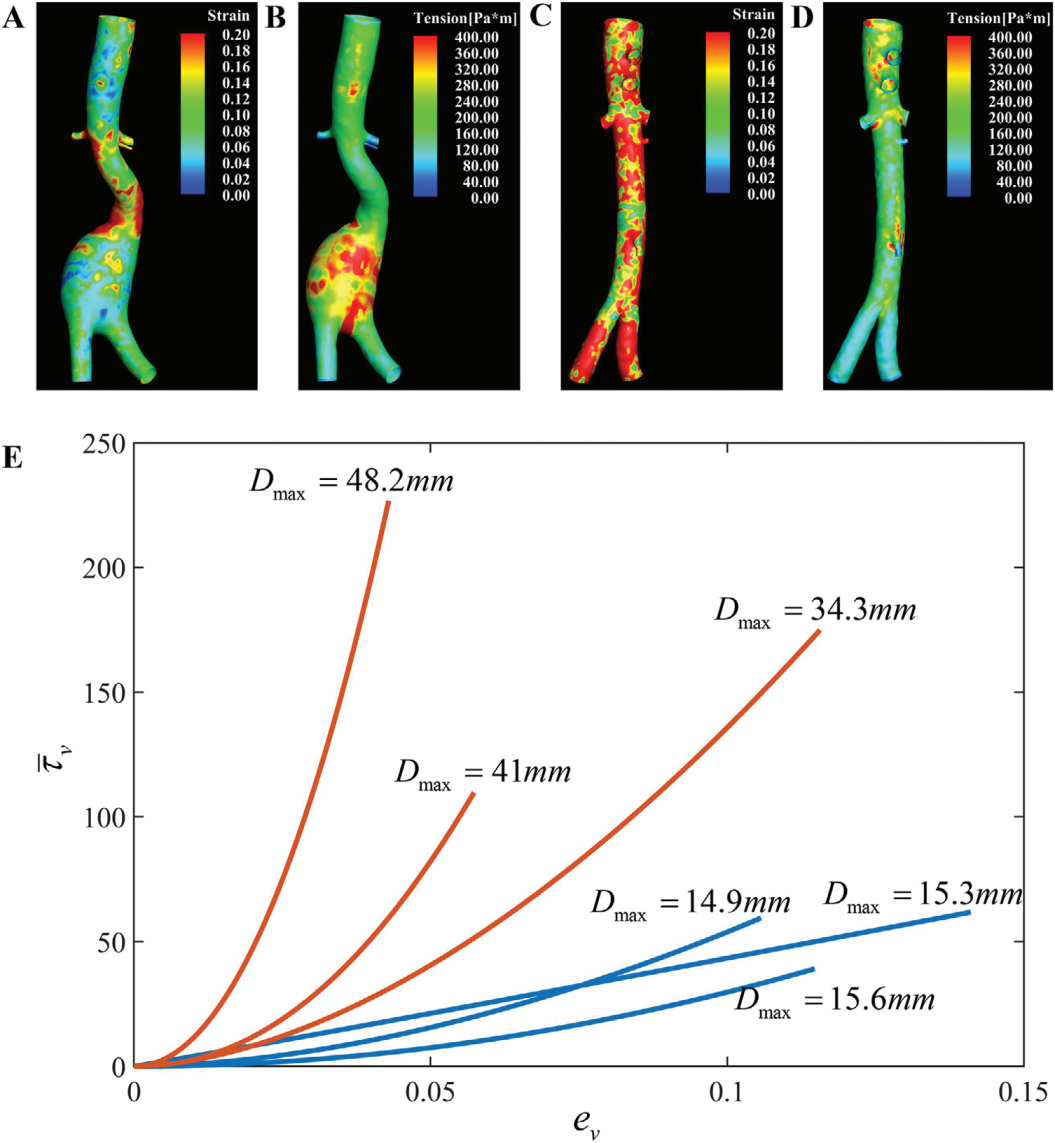
Lumen strain, tension, tension‐strain curves. A,B) Lumen train and lumen tension results of an AAA case (No. 12). C,D) Lumen strain and lumen tension results of a healthy case (No. 2). E) Tension–strain curves and corresponding diameters of six examples, red lines: AAA cases; blue lines: healthy cases.

The lumen geometry and its deformation over cardiac cycle were analyzed to determine the lumen strain. A surrogate membrane model was devised by extruding the lumen surface along the outward normal with a fixed uniform thickness. The wall stress is computed using inverse stress analysis^[^ ^33^, ^37^, ^38^ ^]^ with patient‐specific blood pressures. The distribution of tension and lumen strain at the peak systolic phase are listed in Table S1 (Supporting Information) and two selected examples are presented in **Figure** **3**A–D. It is found that the AAA necks generally bear higher lumen strain than the belly, which agrees with Derwich's conclusion.^[^ ^40^ ^]^ There is no particular concentration in healthy cases for both lumen strain and tension. The AAA cases possess higher lumen strain region on the aneurysm neck, while the lumen strain on the aneurysm bodies trends to be lower than healthy ones. The belly region bears high tension, which was generally higher than that of the healthy aorta.

### 2.4 Regional Tension‐Strain Curves

The challenge of using biomechanical analysis based on medical images, whether CTA, magnetic resonance (MR), or ultrasound, is that the accuracy is limited by the physical resolution of medical images. The maximum or minimum value of strain or tension are susceptible to small variations of segmentation CT images. Therefore, regional averaged value, in which the local uncertainties are somewhat mitigated, was used to describe mechanical characteristics of lumen wall. The region around the maximum diameter plane in AAA cases was generally believed to undergo severest degeneration. The region centered on the maximum diameter plane and 20 mm long along the longitudinal direction was chosen to take the average. A range of 20 mm ensures covering at least 20 contiguous voxels even under the lowest image segmentation requirement of applying the presented method (segmentations no larger than 1 mm in Z direction). The 20 contiguous voxels threshold was proposed to minimize the voxel random noise error in functional MR images and introduced in this work.^[^ ^41^, ^42^ ^]^ The region taking the average in each case is given in Table S1 (Supporting Information), and the regional average tension and lumen strain were computed.

The ensuing tension‐strain curves of all 26 cases are given in Table S1 (Supporting Information). Figure 3E depicts six examples, of which 3 healthy (case No. 1, 2, 3) and 3 aneurysmal (case No. 11, 12, 13), to showcase the comparison of the healthy and aneurysm curves. Red lines are aneurysmal cases, and the blue lines are healthy cases. The nonlinear behavior of arteries is observed. Since our emphasis was on the strain hardening (namely, the change in tissue stiffness over the strain range), the constant term λ_3_ in the tension strain relation is omitted, and only the power part ${\bar{\tau }}_{v}=\lambda_{1}{\left(e_{v}\right)}^{\lambda_{2}}$ is plotted. The fitting coefficients λ_1_, λ_2_ and λ_3_ on the selected region of all cases are listed in Table S2 (Supporting Information).

### 2.5 SSI and dSSI Results

**Figure 4:**
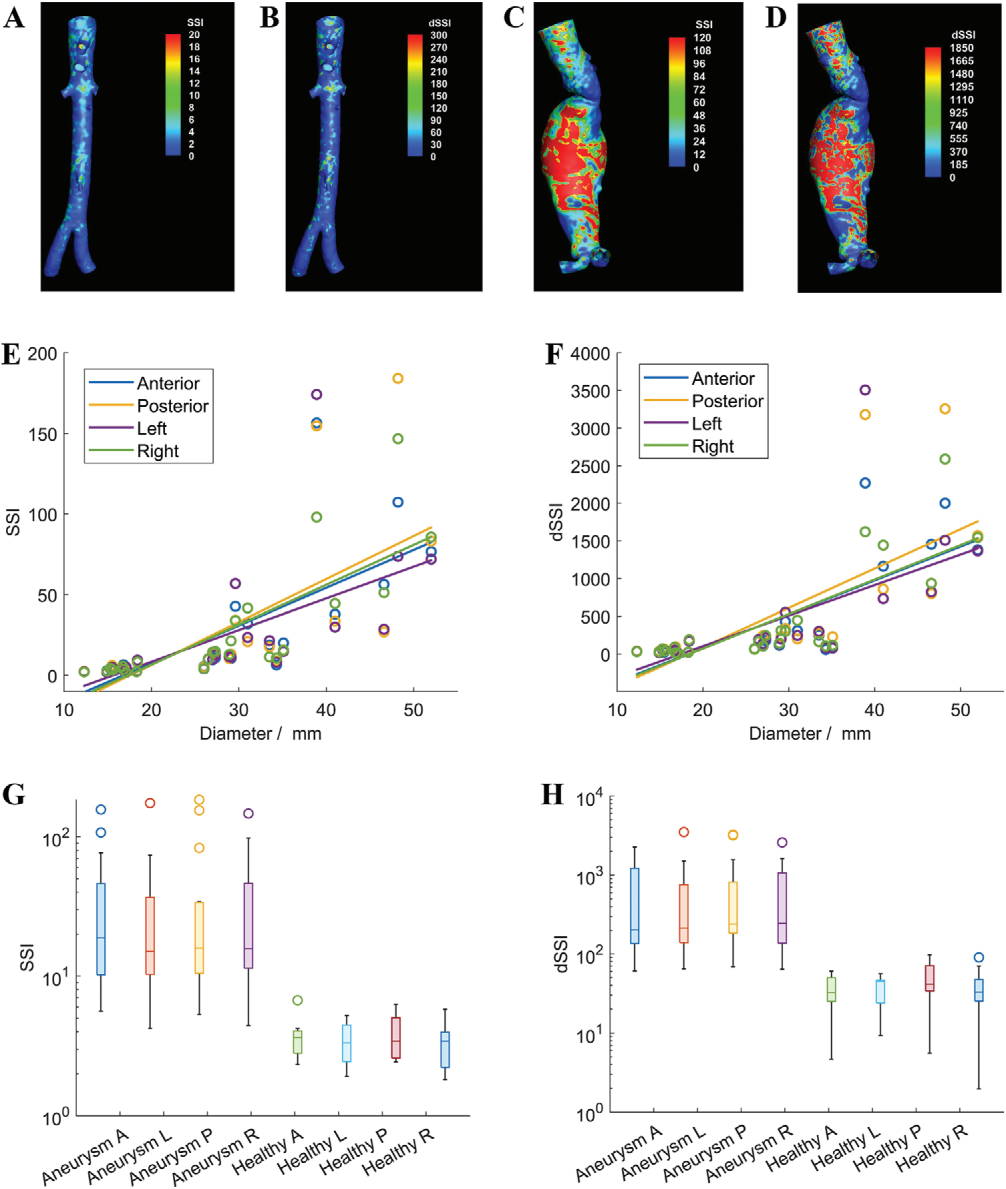
Results of *SSI*, *dSSI*, Pearson correlation and box‐whisker plots. A,B) *SSI* and *dSSI* results of a healthy case (No. 2). C‐D) *SSI* and *dSSI* results of an AAA case (No. 13). E,F) Pearson correlation results of *SSI* and *dSSI* with diameter (n = 26). G,H) Comparison of quadrant *SSI* and *dSSI* between the aneurysm and the healthy groups (n = 26).

Although strain‐tension curves provide valuable information about the mechanical behavior of lumen wall, they can be complex and difficult to be directly interpreted in a clinical setting. Thus, the *SSI* and *dSSI* calculated based on the regional strain tension curves are proposed to further reflect the mechanical lumen properties. The distribution of *SSI* and *dSSI*, including two examples, are presented in **Figure** **4**A–D (the remaining cases are listed in Table S1, Supporting Information). It is noted that the color bar is subject‐specific. The distributions of the healthy cases are relatively uniform and no sharp concentration regions. Both *SSI* and *dSSI* trend to have high values on aneurysm bodies. The difference is significant among different cases, especially for aneurysm cases. It is worthwhile to note that case No. 10 in the aneurysm group was diagnosed as aortic atherosclerosis. Its diameter and morphology appear to be normal, but *SSI* and *dSSI* indicate anomaly.

### 2.6 Statistics of SSI and dSS, and Correlation with Diameter

More statistical analysis was performed to discuss the capability of *SSI* and *dSSI*. Table S2 (Supporting Information) summarizes patient gender, age aneurysm diameter (aortic diameter for healthy group), blood pressure, strain‐tension curve fitting coefficients, and quadrant‐averaged *SSI* and *dSSI*. The linear relationship of *SSI* and *dSSI* with aneurysm/aortic diameters is presented in Figure 4E,F. Both *SSI* (ρ < 0.01) and *dSSI* (ρ < 0.01) of all quadrants are positively correlated to the diameter. The quadrant results are also summarized into box‐whisker plots as shown in Figure 4G,H. The corresponding medians *SSI* and *dSSI* of each group are listed in **Table** **1**.

**Table 1:** Median *SSI* and *dSSI* of all quadrants for different groups. (A,L,P,R) stand for the four quadrants delineated in Figure 8B. A: anterior; L:left, R:right; P: posterior.

Index	Healthy Group (n = 9)				Aneurysm Group (n = 17)			
	A	L	P	R	A	L	P	R
Median *SSI*	3.6	3.3	3.4	3.4	18.8	15.0	15.9	15.7
Median *dSSI*	32.5	44.9	41.2	32.7	203.0	213.5	239.4	244.6

The Pearson correlation analysis shows that both *SSI* and *dSSI* are significantly positively correlated to the aneurysm diameter, as shown in Figure 4E,F. The correlation of *SSI* and *dSSI* with aneurysm diameter indicates that *SSI* and *dSSI* are consistent with the diameter criterion, which is the “golden criterion” in clinical practice. However, the diameter criterion is more statistical and it could ignore some important patient‐specific information. For example, in case No. 11 and case No. 18, the diameters are close but *SSI* and *dSSI* values are far apart. Although it is unclear what makes the difference in *SSI* and *dSSI*, these two cases highlight the possibility of discriminating lesions of similar diameter when *SSI* and *dSSI* are taken into account. Specifically, *SSI* and *dSSI* embody tissue properties, which adds a new dimension to cases where the diameter and morphology are indistinguishable, as demonstrated also in the aortic atherosclerosis patient (case No. 10).

Statistically, as shown in Table 1, the medians *SSI* and *dSSI* of the healthy group differ from the aneurysm group by nearly an order in magnitude. The difference of *SSI* and *dSSI* in aneurysms with different diameters is up to orders in magnitude. The significant difference provides an opportunity to explore potential supplementary criteria to the maximum diameter criterion for aneurysm progression and rupture risk assessment.

### 2.7 Logistic Regression

**Figure 5:**
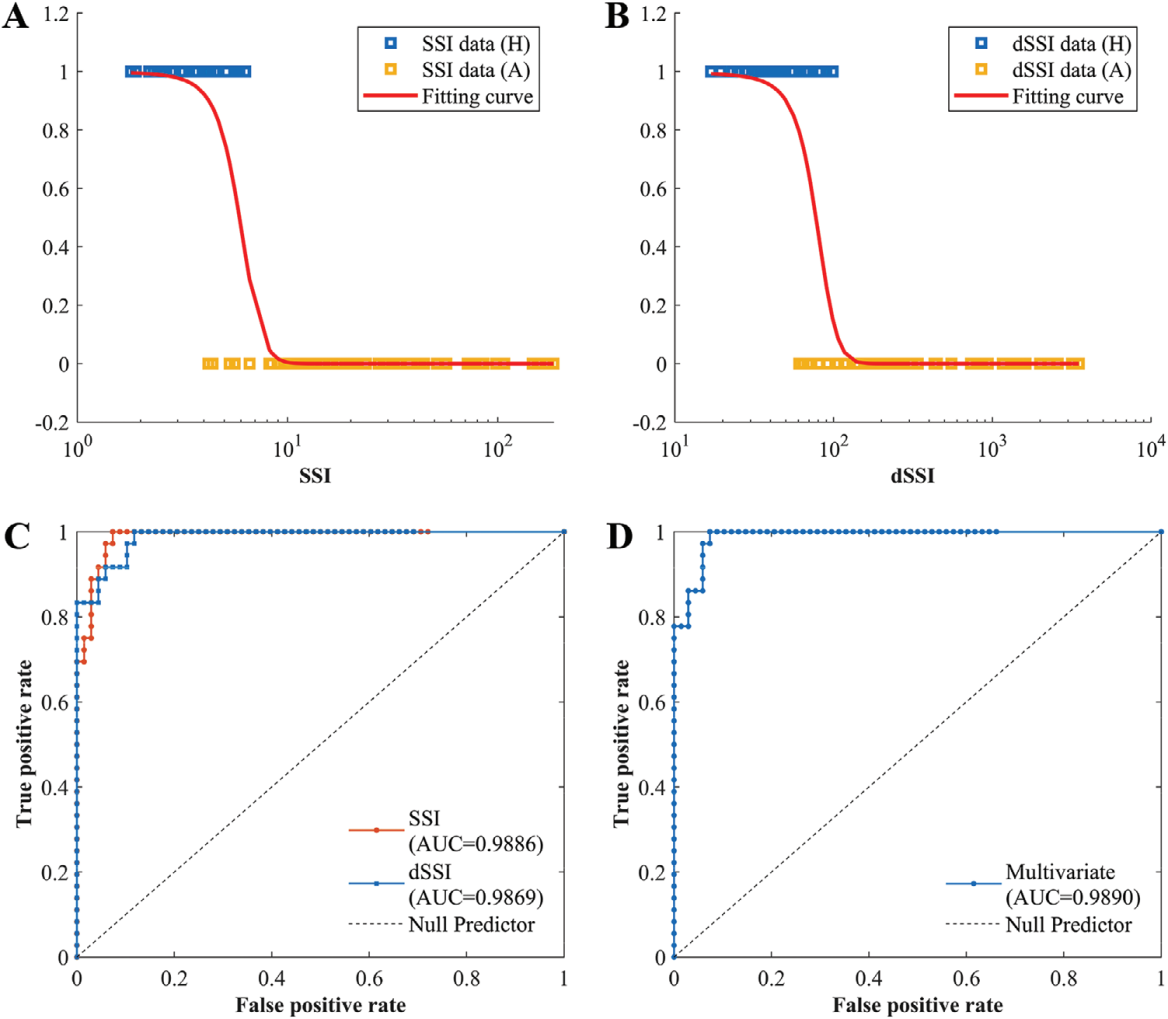
Logistic regression results (n = 26). A) *SSI* model logistic regression curve fitted to data. B) *dSSI* model logistic regression curve fitted to data. C) ROC curves for univariate logistic models. D) ROC curve for multivariate logistic model.

The logistic regression results are summarized in **Table** **2**. It is found that both *SSI* (*p* < 0.01) and *dSSI* (*p* < 0.01) are significant parameters to discriminate healthy and aneurysmal arteries. The fitting curves of the univariate logistic models are presented in **Figure** **5**A,B. Due to the large span in the values of *SSI* and *dSSI*, the x‐axis was set to logarithmic scale. Larger *SSI* and *dSSI* values drive the logistic models to predict aneurysm. The ROC analysis follows. The AUC curves are given in Figure 5C,D. The AUC values of *SSI*, *dSSI*, and their combined model are 0.9886, 0.9869, and 0.9890 respectively, and the corresponding optimal thresholds are given in Table 2.

**Table 2:** Logistic regression results of *SSI* and *dSSI* (n = 26).

Index	*p*‐Value	AUC	Threshold
*SSI*	<0.01	0.9886	0.4450
*dSSI*	<0.01	0.9869	0.7812
Combined	/	0.9890	0.5072

It is noted that the *x*‐axis in Figure 5A,B is logarithmic scale, which means the index value difference between the cases is up to orders in magnitude. In this paper, only binary results, healthy or aneurysmal, are discriminated in the logistic models. However, the large span of these indices, as well as the significant order difference between healthy and aneurysmal wall, provide the possibility of grading the progression of aneurysm based on big data. This may open a new path way for monitoring the progression of the pathological condition from the analyzed mechanical characteristics.

## 3 Discussion

The aim of this paper to advance the clinical application of in vivo biomechanical analysis. The main contributions are twofold: (1) the introduction of a novel in vivo method that makes clinical evaluation of aneurysm mechanical characteristics possible; and (2) the introduction of two new indices, *SSI* and *dSSI*, derived from this method. These indices show orders of magnitude difference between the healthy and the aneurysmal groups. They are positively correlated with diameter (*p* < 0.01), but provide additional information that reflects the aneurysms’ compliance and hardening behavior. The univariate and multivariate logistic regression models based on these indices are capable to sharply discriminate the healthy and the aneurysmal arteries (AUC>0.98 in all models).

The positive correlation with the diameter indicates that the new indices are consistent with the diameter criterion. A larger maximum diameter of an AAA is commonly considered to indicate more severe pathological condition and a higher rupture risk. The tension‐strain curves also show similar shape with tensile test results reported by Wilson and Humphrey,^[^ ^43^ ^]^ where the curve at maximum diameter has higher slope than other regions such as aneurysm neck and non‐aneurysm regions. Healthy cases generally present larger strain amplitude than AAA cases. All AAA cases show higher tension than healthy cases.

The *SSI* values of the healthy group are significantly lower than those of the aneurysm group. This finding is consistent with the known fact the healthy vascular wall possesses a lower stiffness and a larger strain capacity.^[^ ^3^, ^4^, ^32^, ^44^ ^]^ The fiber structure of tissue was investigated microscopically in many studies, see for instance.^[^ ^10^, ^39^, ^45^ ^]^ The elastin fibers of healthy vascular wall are more circumferential oriented and wavier. Aneurysmal tissues undergo remodeling caused by various factors.^[^ ^46^ ^]^ The orientation, waviness as well as the volume fraction of fibers change during remodeling, resulting in a collagen dense network with less distinct layer‐structure, manifested in a higher stiffness.^[^ ^45^ ^]^ The *dSSI* values of the healthy group are also significantly lower than these of the aneurysm group. This indicates that AAAs have a faster pace of strain hardening (in stress‐strain curve, this is manifested by a steeper turn in the J‐shape). The result, again, is consistent with recent studies on the rupture pattern of ascending aortic aneurysms which showed the rupture propensity is strongly correlated with a higher curvature in the stress‐strain curve.^[^ ^11^, ^47^, ^48^ ^]^

AAA diameter and growing rate are still the dominating criteria for surgical intervention. Mechanical and hemodynamic parameters, such as wall shear stress (WSS), wall stress, and strain et al., have been widely investigated in an attempt to enrich the set of indicators for aneurysm rupture risk assessment.^[^ ^13^, ^49^, ^50^ ^]^ These mechanical and hemodynamic parameters are more indicative of pulsating blood pressure, overlooking mechanical behavior of vascular wall. As alluded earlier, true 3D stress analysis is challenging due to the lack of information of wall/ILT geometry. ILT alters wall stress distributions and mechanobiological processes in the wall, which can affect aneurysm expansion and rupture. In the present work, the wall/ILT compound is lumped into a “membrane”, the membrane tension necessary to balance the luminal pressure is computed from equilibrium. We are interested in how the “membrane tension” evolves during lumen deformation. We believe that this embodies the collective stiffness of the wall/ILT compound, which may provide information about pathology and eventually the rupture risk. The two indices, derived along this line of thinking, indeed show the capability to sharply discriminate aneurysm and healthy aorta.

While promising, further validation is still needed. Additional studies with larger, more diverse patient cohorts are required to confirm the utility of *SSI* and *dSSI* for discriminating aneurysm severity and rupture risk. Reproducibility across imaging centers and protocols should also be evaluated. Although we assume that the methodology proposed in the paper is applicable in the presence of thrombus, further research is needed to investigate the effect of thrombus on the mechanical behavior of the vascular wall and to develop more accurate methods for estimating the mechanical behavior of the vascular wall in the presence of thrombus. Furthermore, the underlying biological and biomechanical mechanisms that link tissue mechanics to aneurysm progression are still not fully understood. Integrating knowledge from basic science studies on aneurysm pathophysiology could help refine and improve these indices.

Other limitations should be noted. First, the healthy cases of this study are not truly healthy volunteers. This is a retrospective study. The patients with non‐AAA aneurysms and underwent MD CT scan were included as control group in this study. Although their abdominal aortic morphology may look normal, potential bias could still be introduced to the healthy group. Second, the regional averaged values are prompted in this study, however, the optimal region size is not carefully explored. This will be part of our future work, along with prospective studies and experimental validation of the calculated mechanical characteristics.

While the limitations remain, this work represents an important step toward developing clinically useful biomechanical indicators for patient‐specific AAA risk assessment and decision‐making. With further validation and investigation, these mechanical indices may one‐day aid clinical diagnosis and guide surgical planning beyond what maximal diameter alone can offer. This could lead to improved patient outcomes and reduced AAA ruptures.

## 4 Experimental Section

### Materials

A set of MD CTA images from 26 subjects were included in this study under the approval of the Ethics Committee of Union Hospital, Tongji Medical College, Huazhong University of Science and Technology. The ECG‐gated CTA images captured 34 phases (numbered from 0 to 33) with uniform time interval within an R‐R interval,^[^ ^51^ ^]^ where each phase step represented 3% of an R‐R interval. The CTA images were obtained with a 3rd generation DSCT system equipped with two x‐ray tubes (SOMATOM Force, Siemens Healthcare, Forchheim, Germany). The scanning protocol were as follows: detector collimation 192 mm × 0.6 mm, gantry rotation 0.25 s, pitch from 0.20 to 0.25 according to heart rate, 512 × 512 pixel matrix size, with the field of view for reconstruction 150 mm × 150 mm placed within the abdominal aorta at the level of the renal artery. The voxel sizes were set to 0.4 mm × 0.4 mm in the X‐Y plane and 0.3 mm in the Z direction. The detailed patient characteristics are listed in Table S2 (Supporting Information).

### Methods

**Figure 6:**
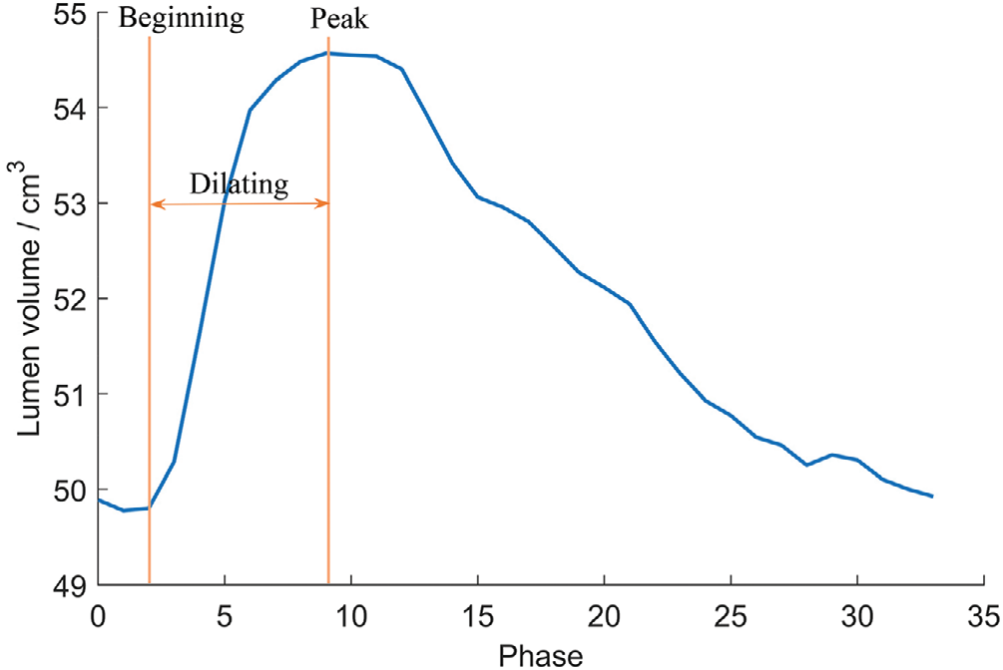
Lumen volume in an R‐R interval (case No. 3).

*Non‐Rigid Image Registration*: To accurately track the voxels displacement in CT images during dilation phases, the diffeomorphic Demons algorithm^[^ ^52^ ^]^ was employed for non‐rigid image registration. A reference phase was selected among the 34 phases, and the corresponding images were denoted as *I_F_*. In this study, the reference phase was generally set to the first phase in an R‐R interval,^[^ ^51^ ^]^ i.e., 0 phase. The dilating phases were determined based on the volume of mesh models, as shown in **Figure** **6**. A dilation process encompassed the phases from the beginning to the peak systole. All the phases between these two were taken as moving phases, and the images were denoted as *I_M_*. The dilation process contained 6–9 phases, which varied across different patients.

The images of dilation phases were registered to the reference phase. In diffeomorphic Demons algorithm, for a given moving image *I_M_* and a reference image *I_F_*, a transformation *T* was needed to minimize the cost function *M*(*T*(*I_M_*), *I_F_*), which was given as^[^ ^53^ ^]^

(1) $$\min\left(M\left(T\left(I_{M}\right),I_{F}\right)\right)$$

In this work, Mutual Information was chosen as the cost function and the symmetric image normalization method (SyN)^[^ ^54^ ^]^ was chosen as the transformation. Newton‐Rephson iteration method was used in computing the transformation *T*.

The Dice score was used to evaluate the quality of the registration process, which is a measure of the similarity between the aortic wall contour of the reference and moving phases, and is calculated using the following equation:

(2) $$Dice\left(I_{F},I_{M}\right)=\frac{2\left|I_{F}\cap I_{M}\right|}{\left|I_{F}\right|+\left|I_{M}\right|}$$

A Dice score closer to 1 indicates better registration performance.

*Aortic Lumen Wall Reconstruction*: An arterial lumen model was extracted from CTA medical images at the reference phase. Based on the model, a 3D surface mesh was reconstructed with Insight Toolkit (ITK), which was used as a surrogate model by treating the surrounding material (wall/thrombus) as an elastic membrane. The initial mesh generated by ITK was found to be relatively rough, and to address this, Laplace smoothing was employed to achieve a smoother mesh.^[^ ^55^ ^]^ A thin layer of solid prism elements was then constructed to represent the thin lumen wall by extruding the surface mesh model along the outward normal direction. The lumen thickness was uniformly set to 0.01 mm and the element size of the aneurysm models was no larger than 0.8mm.

**Figure 7:**
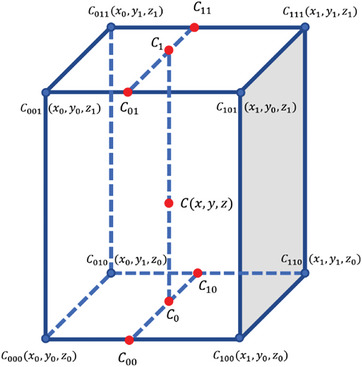
Depiction of 3D interpolation.

A deforming mesh was obtained by applying the node displacement of each phase to the reference mesh. The trilinear interpolation techniques were used to calculate node displacement from the voxel displacement of voxel. For a given interpolation node point *C*(*x*, *y*, *z*), as shown in **Figure** **7**, its eight nearest voxel corner points *C*_000_, *C*_001_, *C*_010_, *C*_011_, *C*_100_, *C*_101_, *C*_110_ and *C*_111_ enclosing *C* were identified, whose displacements were known from registration. The coordinates of *C* after deformation, *C*′(*x*′, *y*′, *z*′) was the objective to be determined through interpolation. Let *x_d_*, *y_d_*, and *z_d_* be the differences between each of x, y, z and the smaller coordinate related, that is:

(3) $$x_{d}=\frac{x-x_{0}}{x_{1}-x_{0}}$$

(4) $$y_{d}=\frac{y-y_{0}}{y_{1}-y_{0}}$$

(5) $$z_{d}=\frac{z-z_{0}}{z_{1}-z_{0}}$$

The displacement vector ${\vec{D}}_{C_{mn}}$ of *C*_00_, *C*_01_, *C*_10_, *C*_11_ could be calculated as:

(6) $${\vec{D}}_{C_{mn}}=\left(1-x_{d}\right){\vec{D}}_{C_{0mn}}+x_{d}{\vec{D}}_{C_{1mn}}$$

where (*m*, *n* = 0or1). Further, the displacement vector ${\vec{D}}_{C_{n}}$ of *C*_0_, *C*_1_were calculated as:

(7) $${\vec{D}}_{C_{n}}=\left(1-y_{d}\right){\vec{D}}_{C_{0n}}+y_{d}{\vec{D}}_{C_{1n}}$$

Finally, the voxel displacement vector ${\vec{D}}_{C}$ of voxel *C* was determined as below:

(8) $${\vec{D}}_{C}=\left(1-z_{d}\right){\vec{D}}_{C_{0}}+z_{d}{\vec{D}}_{C_{1}}$$

Thus, the coordinates of *C*′ could be expressed as

(9) $$C^{\prime }\left(x^{\prime },y^{\prime },z^{\prime }\right)=C\left(x,y,z\right)+{\vec{D}}_{C}$$

where *C*(*x*, *y*, *z*) was the corresponding of node *C*′ on the reference mesh.

*Lumen Strain and Tension Analysis*: Lumen strain was calculated from the displacement obtained from registration. The Green–Lagrange strain tensor is defined as

(10) $$E=\frac{1}{2}\left(F^{T}\cdot F-I\right)=\left[\begin{array}{ccc} e_{11} & e_{12} & e_{13}\\ e_{21} & e_{22} & e_{23}\\ e_{31} & e_{32} & e_{33} \end{array}\right]$$

The equivalent strain was used in the succeeding analysis:

(11) $$\begin{array}{ccc} e_{v} & = & \sqrt{\frac{1}{2}}\left({\left(e_{11}-e_{22}\right)}^{2}+{\left(e_{33}-e_{11}\right)}^{2}+{\left(e_{33}-e_{22}\right)}^{2}\right.\\ & & +\;\left.\right.{\left.6\times \left({\left(e_{12}\right)}^{2}+{\left(e_{23}\right)}^{2}+{\left(e_{13}\right)}^{2}\right)\right)}^{1/2} \end{array}$$

The equivalent strain depicts an envelope of the direct and shear components. It was comprehensive measure of strain level. It is zero in an equal‐triaxial strain state, but this case is irrelevant to the application.

As there is no wall geometry data to support 3D stress analysis, the study uses a surrogate model that treats the surrounding material (wall/thrombus) as an elastic membrane. The “membrane tension” can be reliably predicted using inverse stress analysis. This approach is shown to be effective tension/stress analysis in aneurysm problems.^[^ ^33^, ^34^, ^35^, ^36^, ^37^, ^38^ ^]^ iFEM uses the same work form as in the forward finite element

(12) $$\delta \Pi \left(u\right)≔\int_{\Omega_{0}}\delta F:\mathrm{FS}dV-\langle f_{ext},\delta u\rangle =0$$

where $\delta u\in u:{\delta u|}_{\partial \Omega_{u}}=0$, was a kinematically admissible variation of displacement, Gradφ the deformation gradient, and **S** the second‐Piola‐Kirchhoff stress. The ensuing FEM equation is used to find the inverse deformation that brings the current geometry back to a reference configuration.^[^ ^28^ ^]^ For the surrogate thin lumen model, the membrane tension is statically determined and hence can be estimated without using the realistic elastic properties of the tissue by the so‐called inverse stress/tension analysis that directly solves tension distribution in the imaged geometry.^[^ ^37^, ^56^, ^57^ ^]^ When the stress was obtained, the tension was uniformly computed using

(13) $$\tau_{v}=h\cdot \sigma_{v}$$

where *h* was the thickness that was uniformed applied to all cases. The equivalent tension τ_*v*_ was calculated with the same form of Equation (11).

Based on the principal of static determinacy, Miller et al used simple linear stress–strain relation of high stiffness to compute the wall tension, and found that the results agreed well with those obtained from sophisticated nonlinear constitutive laws.^[^ ^58^ ^]^ Thus, a Neo‐Hookean model with density of 1050 kg m^−^3, Poisson's ratio of 0.45, and Young's modulus of 2 × 10^5^ MPa was used in the iFEM analysis. Patient‐specific diastolic blood pressure and systolic blood pressure recorded before MD CTA data collecting were used in tension analysis. This pressure varied as the dilating phases and was uniformly applied to the lumen wall at each phase.

*Sensitivity Study*: Uniform lumen wall thickness was assumed in the tension analysis. To test the sensitivity of lumen thickness on tension, the six example models were selected to extrude two different thicknesses of 0.1 mm and 0.01 mm. The average error over the whole models is no more than 2.5% both for strain and tension, as listed in Table S3 (Supporting Information). Though it has been demonstrated that the tension predictions are insensitive to the material behavior in iFEM analysis,^[^ ^37^ ^]^ the study tested an example case with different material models in the inverse tension analysis, a linear isotropic elastic, a nonlinear isotropic(Neo‐Hookean) and a nonlinear anisotropic (Gasser‐Ogden‐Holzapfel model, GOH) are employed. The maximum difference is <1% when comparing to the result of linear isotropic model. The detailed setting information and results are given in the Table S4 (Supporting Information).

*Tension—Strain Curve*: As an intermediate step, the “tension” is fitted to a function of the strain. A power law with a constant was used to relate equivalent lumen tensionτ_*v*_ to the equivalent lumen strain *e_v_*:

(14) $$\tau_{v}=\lambda_{1}{\left(e_{v}\right)}^{\lambda_{2}}+\lambda_{3}$$

where the coefficients λ_1_ > 0, λ_2_ > 1 and λ_3_ > 0. These parameters were determined by a best‐fit approach. λ_3_ was introduced to cover the residual tension, which is zero in controlled ex vivo tensile test.^[^ ^59^ ^]^

**Figure 8:**
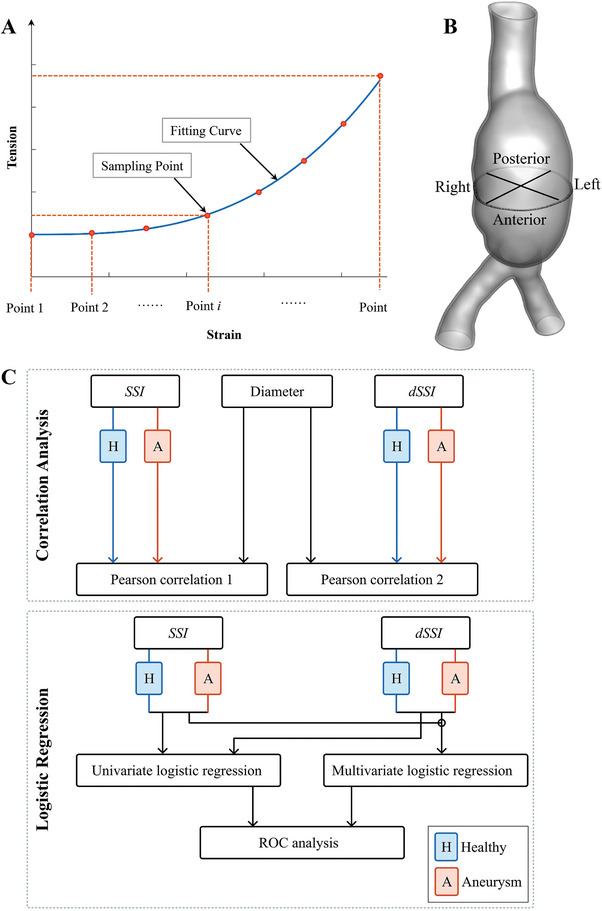
Method depictions. A) Tension–strain curve fitted from each phase in dilation range. B) Depiction of quadrant division. C) Workflow of statistical analysis.

*Strain‐Scaled Stiffness Index and Hardening Index*: To describe the stiffness of wall structure, two indices, namely the scaled stiffness index (*SSI*) and the corresponding hardening index *dSSI*, were proposed. The indices were defined as:

(15) $$SSI=\frac{1}{e_{\max}}\frac{1}{p_{s}}mean\left(\frac{\partial \tau_{v}\left(e_{v}\right)}{\partial e_{v}}\right)=\frac{1}{e_{\max}}\frac{1}{p_{s}}\frac{1}{N}\sum_{i=1}^{N}\lambda_{1}\lambda_{2}e_{i}^{\lambda_{2}-1}$$

(16) $$\begin{array}{c} dSSI=\frac{1}{e_{\max}}\frac{1}{p_{s}}mean\left(\frac{\partial^{2}\tau_{v}\left(e_{v}\right)}{\partial {e_{v}}^{2}}\right)=\frac{1}{e_{\max}}\frac{1}{p_{s}}\frac{1}{N}\sum_{i=1}^{N}\lambda_{1}\lambda_{2}\left(\lambda_{2}-1\right)e_{i}^{\lambda_{2}-2} \end{array}$$

where *e*_max _ was the strain amplitude at end‐systolic phase, *p_s_* was the patient‐specific systolic pressure, *N* was the total number of points sampled on the fitted curve, *e_i_* was the strain of *i* ^th^ sampling point, as shown in **Figure** **8**A. Numerical tests showed that when *N* ≥ 16, the variation of the *SSI* and *dSSI* values would not exceed 5% when *N* was increased. Thus, *N* was set to 16 in this study.

The two indices, *SSI* and *dSSI*, were both consisted of three elements. The first element was the reciprocal of the lumen strain amplitude. This component is important as the physiological strain range of aneurysms is generally smaller than that of healthy tissue. By itself, the strain range could be a meaning indicator. The second element was the reciprocal of patient‐specific systolic pressure, which was introduced mainly to nondimensionalize these indices. The third component was the average stiffness or its hardening rate. Numerous studies had established a relationship between the aneurysmal degeneration (or rupture risk) and strain amplitude, with an understanding that the strain amplitude decreased in AAA patients.^[^ ^4^, ^32^, ^44^ ^]^ However, the tension–strain curve of the aortic wall exhibited high nonlinearity, with significant strain hardening. The stiffness at a particular phase doesn't provide a comprehensive representation of the mechanical characteristics of aneurysm. In contrast, the averages over the entire strain range provide a better representation of the stiffness and hardening rate. In Equations (15) and (16) the strain amplitude was aggravated with the mean stiffness and mean hardening rate to amplify the variation of these indices.

*Statistical Methods*: Maximum or minimum value of strain and tension on the model may be influenced by small variation in the segmentation of CT images, despite efforts to reconstruct the geometric models as accurately and consistently as possible. This was also true for *SSI* and *dSSI*. Therefore, regional averages were used in the statistical analysis. For the aneurysm cases (n = 17), it was found that the high value region of *SSI* and *dSSI* was generally consistent with the region of maximum aneurysm diameter. The segment centered in the max diameter plane and 20 mm long along the longitudinal direction was chosen to take the average. For the healthy cases (n = 9), the difference among different regions of abdominal aorta was minor and a subsection of 20 mm segment centered at the middle cross section was chosen to take the average. All the models are listed in the Table S1 (Supporting Information) with the analyzed subsection highlighted on the model.

To accommodate heterogeneity of wall tissue, which was reported in several publications,^[^ ^30^, ^46^, ^59^, ^60^, ^61^ ^]^ the segments were further divided into four quadrants, namely left, right, anterior, and posterior as depicted in Figure 8B. These four quadrants were treated as independent groups, and noted as group L, group R, group A and group P respectively. The box‐whisker plot was used to quantitatively compare the quadrants in each group, and further applied in the following statistical analysis.

Pearson correlation analysis was conducted to evaluate the linear correlations of *SSI* and *dSSI* with diameters, which is the golden criterion in present clinical practice. The above statistical analyses were depicted in Figure 8C and performed using IBM SPSS Statistics (IBM Inc., Armonk, New York), with statistical significance set at *p* < 0.05 in all cases.

The availability of *SSI* and *dSSI*, which are continuous variables varying from 0 to infinite, begs the question as to whether there exist thresholds for *SSI* and *dSSI* that can optimally distinguish healthy and aneurysm wall tissues. To address this question, logistic regression was employed to calculate the probability of the above binary event occurring,^[^ ^62^, ^63^ ^]^ i.e., the evaluated parameters predicting a healthy case or aneurysmal case. Briefly, the standard logistic function was defined as

(17) $$\sigma \left(t\right)=\frac{1}{1+e^{-t}}$$

where σ:R→(0,1), *t* was a linear function of a single or multiple variables. The *t* function could be expressed as

(18) $$t=\beta_{0}+\sum_{i=1}^{N}\beta_{i}x_{i}$$

where *N* = 1 corresponded to a univariate model, and *N* > 1 a multivariate model. Here β_*i*_ were the coefficients to be fitted to the data, and *x_i_* was the observing variable. In this paper, *x_i_* could be *SSI*, *dSSI* or their combination. The quality of fitting was evaluated by the maximum likelihood estimation method to find the best fit.^[^ ^62^ ^]^ Then, the general logistic function p:R→(0,1) could be written as

(19) $$p\left(x\right)=\sigma \left(t\right)=\frac{1}{1+e^{-(\beta_{0}+\sum_{i=1}^{N}\beta_{i}x_{i})}}$$

Finally, the odds were defined as the ratio of the two probabilities (healthy over aneurysmal), and given as

(20) $$odds=\frac{p\left(x\right)}{1-p\left(x\right)}=e^{\beta_{0}+\sum_{i=1}^{N}\beta_{i}x_{i}}$$

The logistic regression analysis aimed to predict whether a region was healthy or aneurysmal using the aforementioned parameters, either separately or combinedly. Healthy and aneurysmal status were defined as categorical variables. Univariate and multivariate logistic regression analysis were conducted. Receiver operating characteristics (ROC) analysis was performed on all logistic models to calculate the area under the ROC curve (AUC), which was used to estimate the capabilities of the logistic models in discriminating healthy and the aneurysmal status. A true positive prediction (TP) was defined as a correctly predicted healthy case, while a false positive prediction (FP) was defined as a predicted healthy case that was actually an aneurysmal case. The negative prediction scenarios were similarly defined. Statistical significance was defined as *p*<0.05. The logistic regression analysis was conducted through MATLAB (The MathWorks Inc, Natick, Mass).

## Conflict of Interest

The authors declare no conflict of interest.

## Author Contributions

T.H., X.Q., and L.C. contributed equally to this work. T.H. and L.C. contributed to research design, computation, and manuscript writing. X.Q. and C.Y. contributed to data collection, supervising, and manuscript revision. M.Y., Z.L., and Q.L. contributed to data collection. H.L. contributed to image processing and registration computation. C.Y., X.Q., and Q.L. contributed to funding acquisition. P.Q. contributed to conceptual development and supervising. Y.L. and J.L. contributed to conceptual development, research design, supervising, and writing. All authors read and approved the final version of the manuscript.

## Supporting information



Supporting Information

## Data Availability

The data that support the findings of this study are available from the corresponding author upon reasonable request.
