# Supplementary material for: Regional Stiffness and Hardening Indices: New Indicators Derived from Multidimensional Dynamic CTA for Aneurysm Risk Assessment
Source: Adv Sci (Weinh). 2024 Oct 25;11(47):2400653. doi: 10.1002/advs.202400653 (PMC11653626; doi:10.1002/advs.202400653)
Supplement: Supplementary file 1 — Supporting Information [file ADVS-11-2400653-s001.pdf]

ADVANCED SCIENCE Open Access

Supporting Information

for *Adv. Sci.*, DOI 10.1002/advs.202400653

Regional Stiffness and Hardening Indices: New Indicators Derived from Multidimensional Dynamic CTA for Aneurysm Risk Assessment

Tianming Huang, Xiaoyu Qi, Lan Cao, Ming Yang, Huan Luo, Qin Li, Peidong Qian, Jia Lu, Ziqiao Lei, Yuanming Luo* and Chao Yang**

Supporting Information

Regional Stiffness and Hardening Indices: New Indicators Derived from Multidimensional Dynamic CTA for Aneurysm Risk Assessment

Tianming Huang[1], *Xiaoyu Qi*[2], *Lan Cao*[1], *Ming Yang*[3], *Huan Luo*[1], *Qin Li*[2], *Peidong Qian*[1], *Jia Lu*[4], *Ziqiao Lei*[3]*, *Yuanming Luo*[4]*, *and Chao Yang*[2]*

I. **Table S1** Lumen model, lumen strain, tension, selected analyzing region, strain-tension curve, *SSI* and *dSSI* of all cases

**Case No.**	Before and after smoothing	Strain	Tension	Selected Region	Strain-tension curve	*SSI*	*dSSI*
1	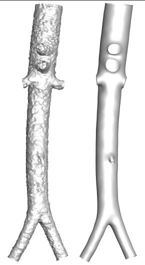	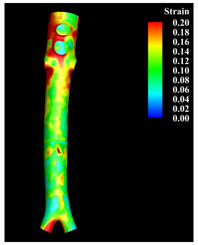	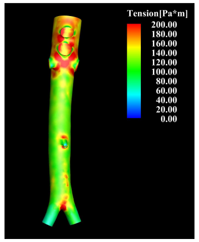	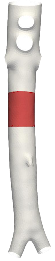	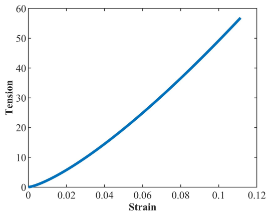	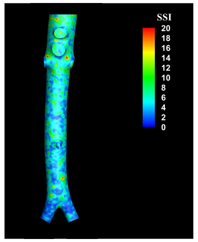	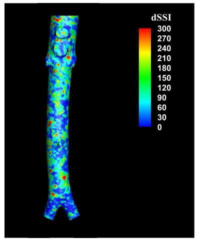
2	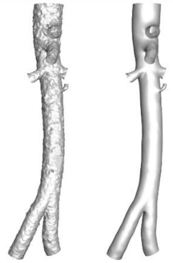	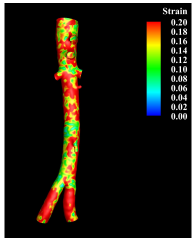	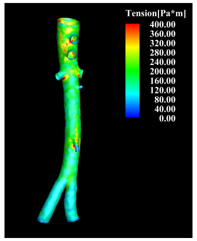	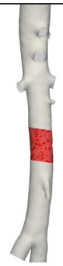	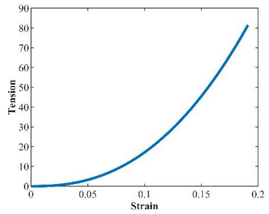	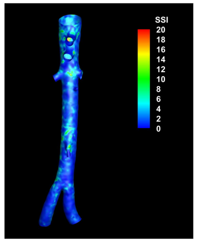	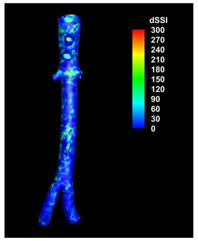

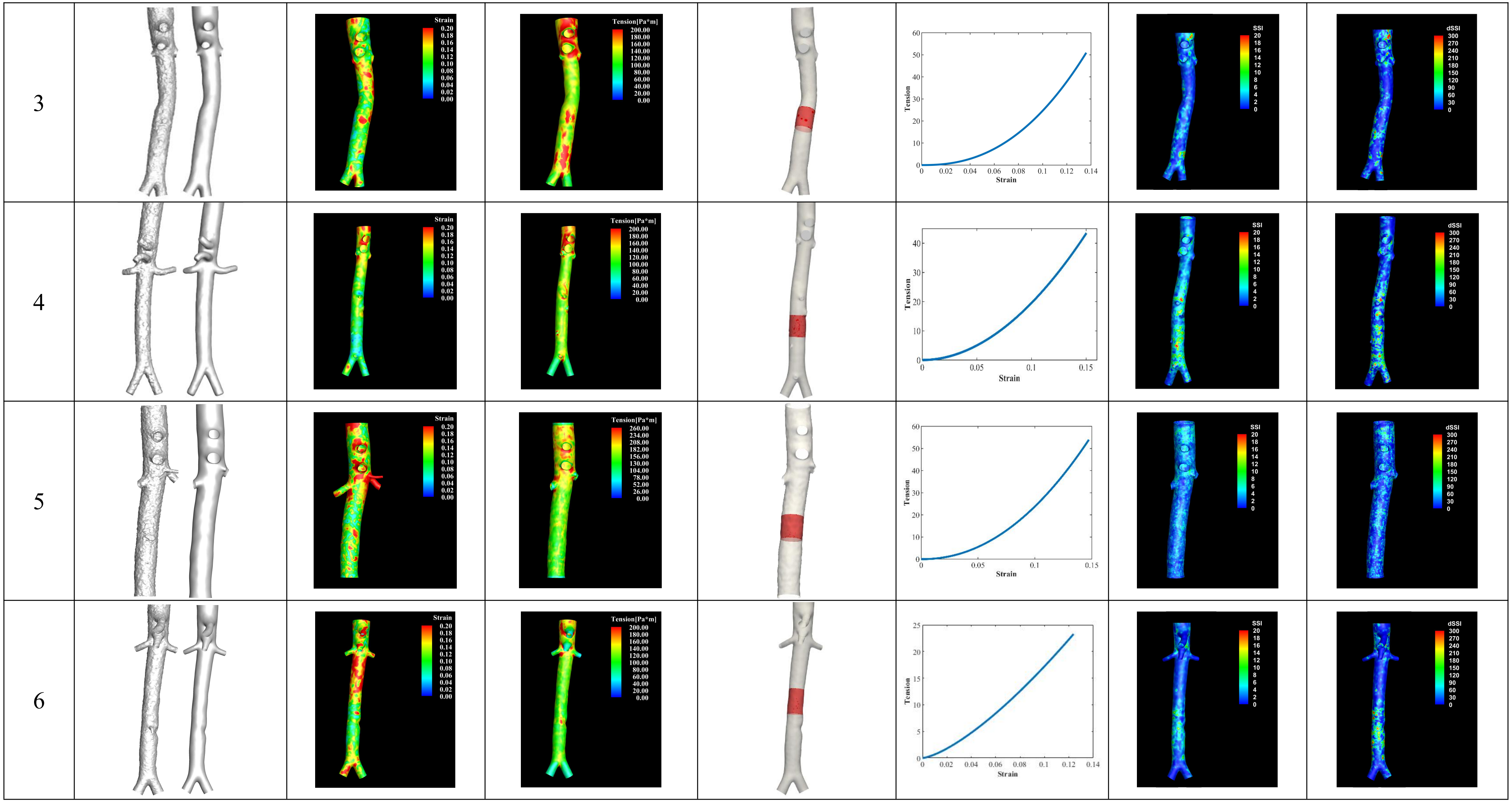
3
4
5
6
Strain
Tension[Pa*m]
Tension
Strain
SSI
dSSI

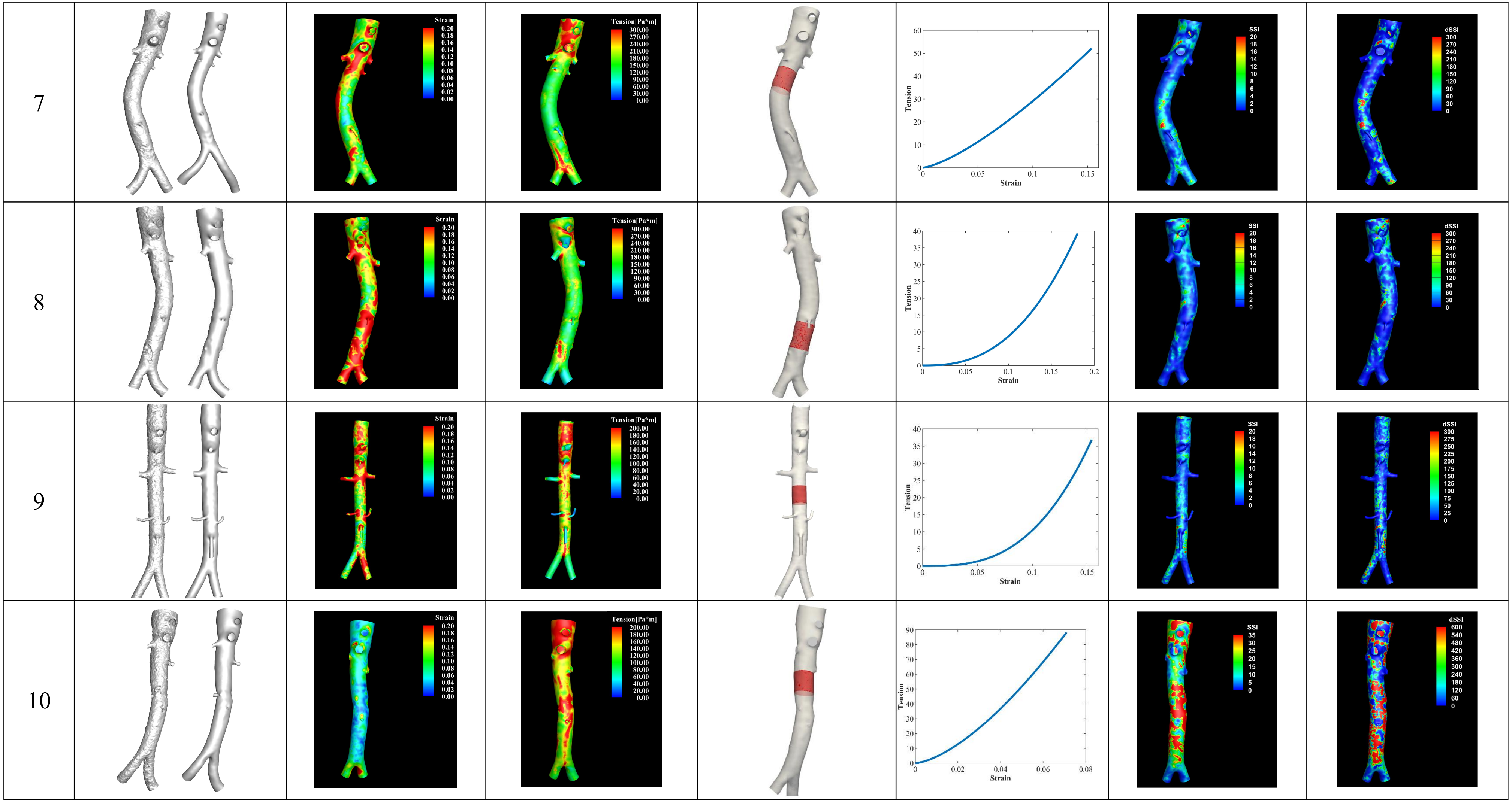
7
8
9
10
Strain
Tension[Pa*m]
Tension
Strain
SSI
dSSI

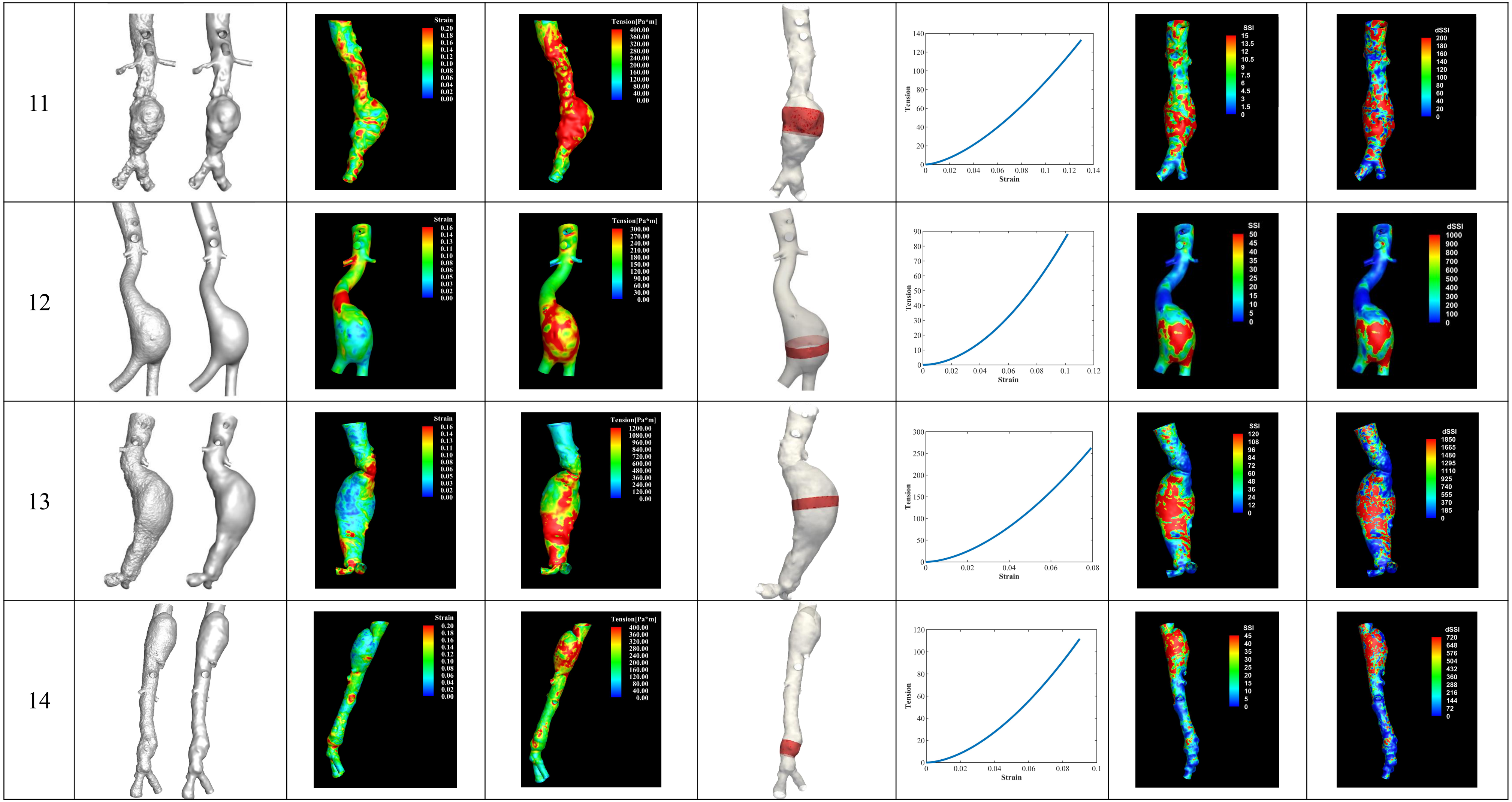
11
12
13
14
Strain
Tension[Pa*m]
Tension
Strain
SSI
dSSI

15	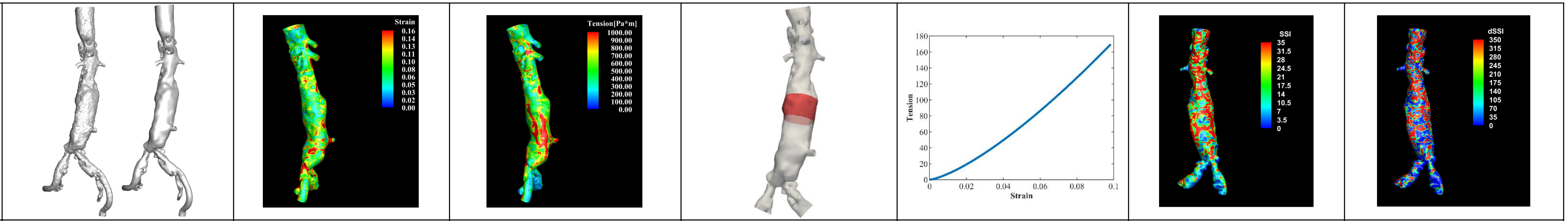
16	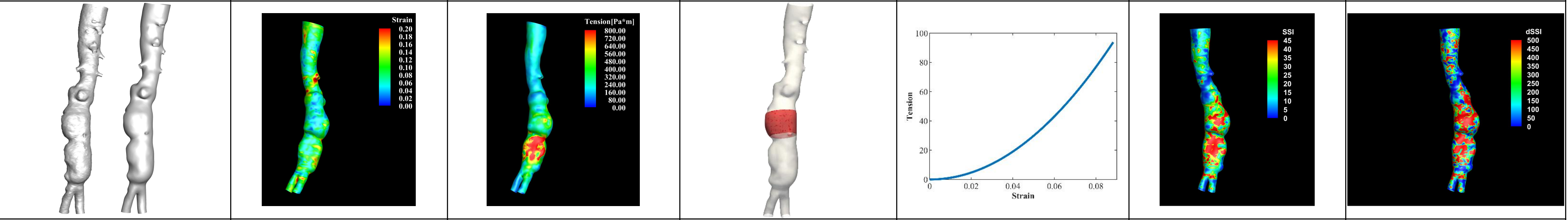
17	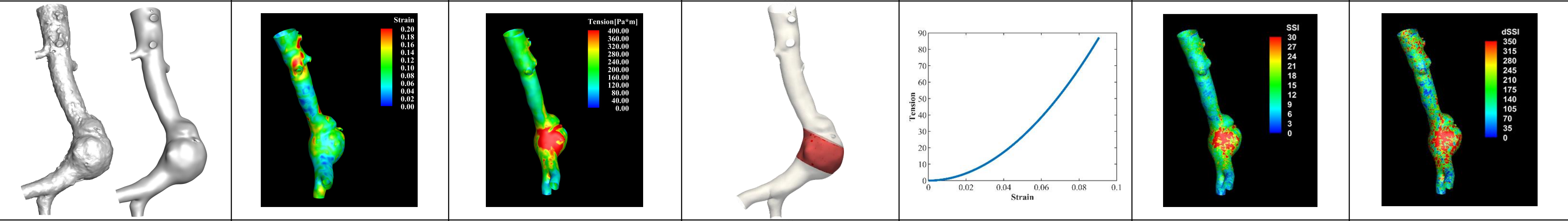
18	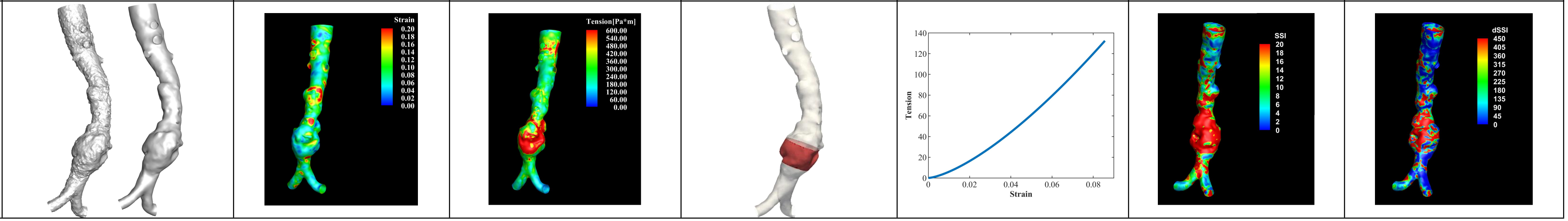

19

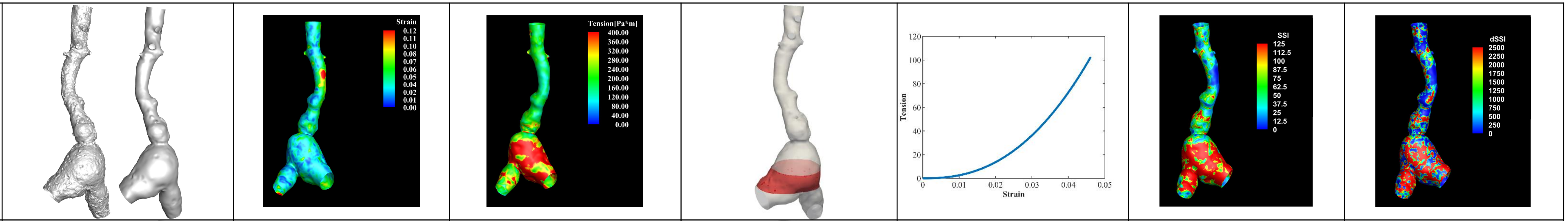
Strain
0.12 0.11 0.10 0.08 0.07 0.06 0.05 0.04 0.02 0.01 0.00
Tension[Pa*m]
400.00 360.00 320.00 280.00 240.00 200.00 160.00 120.00 80.00 40.00 0.00
Tension
Strain
SSI
125 112.5 100 87.5 75 62.5 50 37.5 25 12.5 0
dSSI
2500 2250 2000 1750 1500 1250 1000 750 500 250 0

20

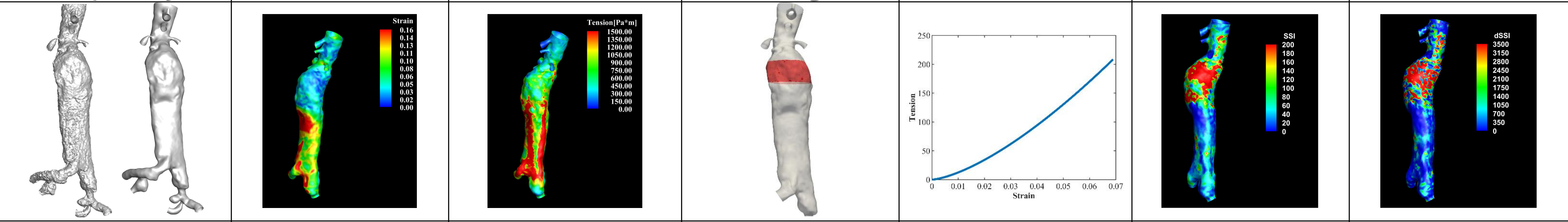
Strain
0.16 0.14 0.13 0.11 0.10 0.08 0.06 0.05 0.03 0.02 0.00
Tension[Pa*m]
1500.00 1350.00 1200.00 1050.00 900.00 750.00 600.00 450.00 300.00 150.00 0.00
Tension
Strain
SSI
200 180 160 140 120 100 80 60 40 20 0
dSSI
3500 3150 2800 2450 2100 1750 1400 1050 700 350 0

21

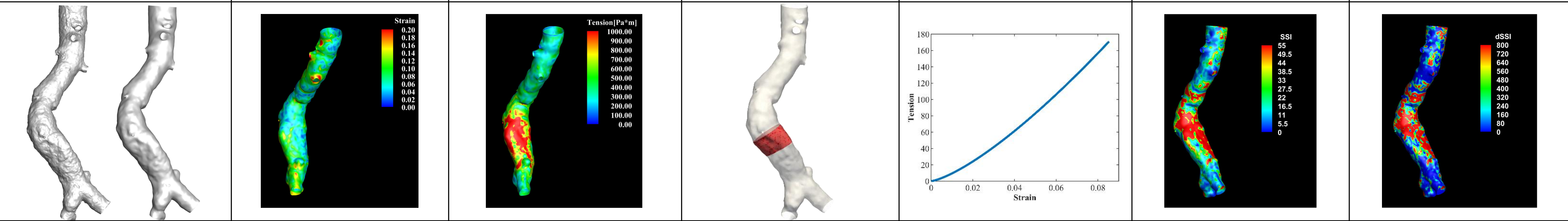
Strain
0.20 0.18 0.16 0.14 0.12 0.10 0.08 0.06 0.04 0.02 0.00
Tension[Pa*m]
1000.00 900.00 800.00 700.00 600.00 500.00 400.00 300.00 200.00 100.00 0.00
Tension
Strain
SSI
55 49.5 44 38.5 33 27.5 22 16.5 11 5.5 0
dSSI
800 720 640 560 480 400 320 240 160 80 0

22

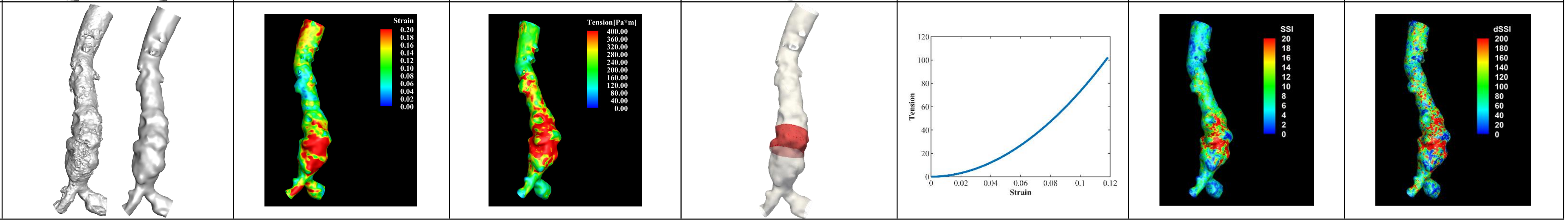
Strain
0.20 0.18 0.16 0.14 0.12 0.10 0.08 0.06 0.04 0.02 0.00
Tension[Pa*m]
400.00 360.00 320.00 280.00 240.00 200.00 160.00 120.00 80.00 40.00 0.00
Tension
Strain
SSI
20 18 16 14 12 10 8 6 4 2 0
dSSI
200 180 160 140 120 100 80 60 40 20 0

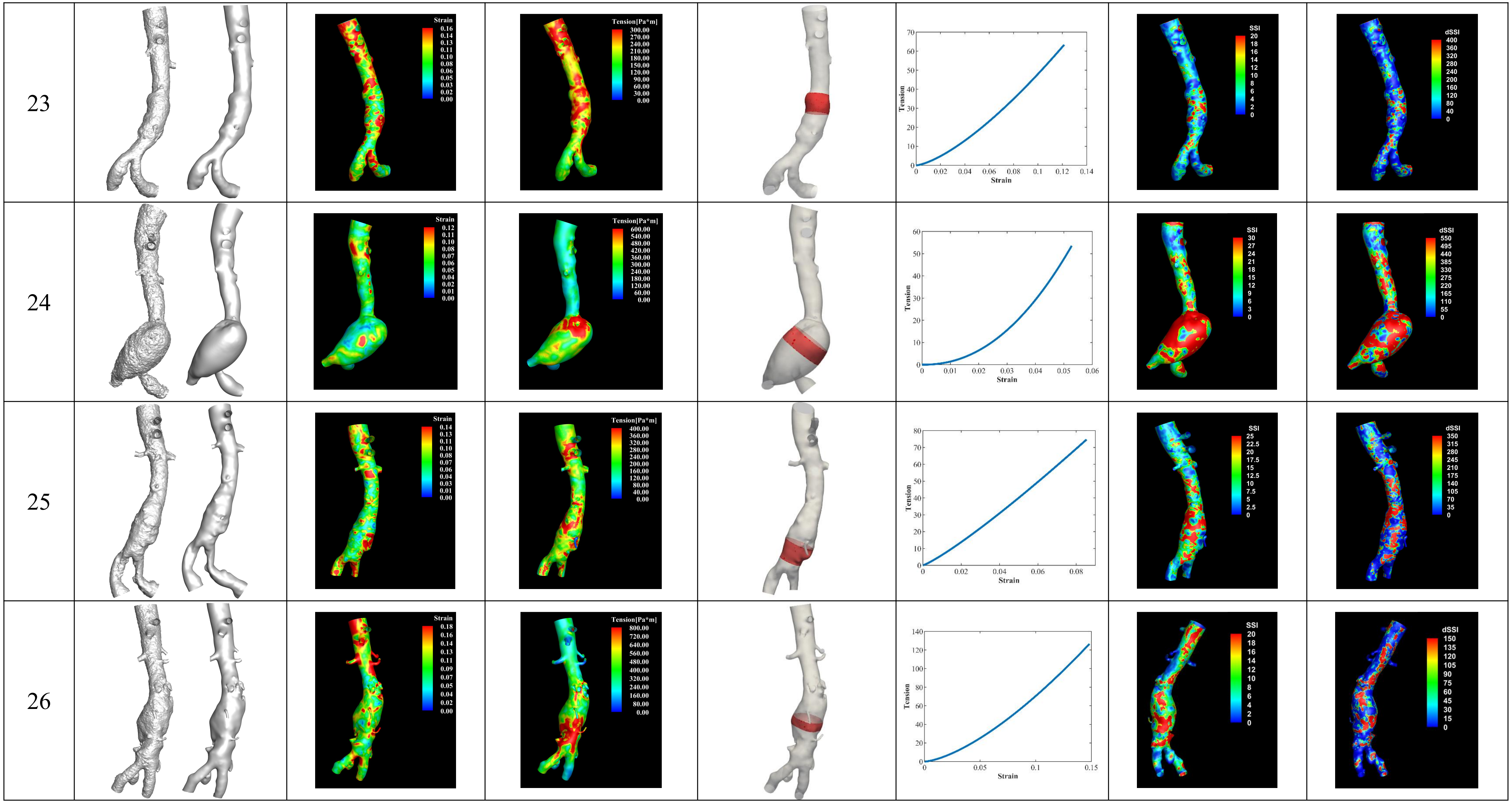

23
24
25
26
Strain
Tension[Pa*m]
Tension
Strain
SSI
dSSI

II. **Table S2** Demographic, diameter, fitting coefficients and quadrant-averaged results

**Case No.**	**Gender**	**Age**	**Pressure (mmHg)**	**Diameter (mm)**	**Group**	λ_1	λ_2	λ_3	**Index**	A	P	L	R
**1**	F	31	142/65	15.3	H	1058.39	1.33	0.11	*SSI*	4.2	4.5	4.2	4.5
									dSSI	60.5	66.0	56.0	69.9
**2**	M	30	137/75	14.9	H	4382.15	2.41	0.19	*SSI*	2.9	2.6	1.9	2.6
									dSSI	25.1	36.8	19.4	30.6
**3**	M	60	119/79	15.6	H	2714.89	1.93	0.13	*SSI*	3.6	3.4	3.2	3.4
									dSSI	48.1	41.2	44.9	39.8
**4**	M	42	117/78	15.5	H	1003.33	1.30	0.14	*SSI*	4.0	6.3	5.1	3.7
									dSSI	24.8	66.6	45.8	35.6
**5**	o	o	134/92	18.3	H	3040.15	2.11	0.15	*SSI*	2.3	2.4	2.5	2.2
									dSSI	27.6	25.0	25.4	27.8
**6**	M	64	137/75	12.3	H	441.67	1.41	0.12	*SSI*	2.4	2.5	2.4	2.2
									dSSI	32.5	39.3	34.6	32.7
**7**	M	60	130/86	16.6	H	667.47	1.36	0.15	*SSI*	3.6	4.7	5.9	2.8
									dSSI	36.2	98.5	79.8	49.7
**8**	F	59	148/84	17.1	H	3167.68	2.56	0.181	*SSI*	1.8	3.4	2.5	3.2
									dSSI	16.9	38.2	31.5	23.1
**9**	M	41	128/85	16.7	H	8677.21	2.92	0.15	*SSI*	3.4	5.6	3.7	5.8
									dSSI	44.1	83.1	49.5	90.1
**10**	M	72	171/110	18.4	A	4991.39	1.53	0.07	*SSI*	9.2	9.0	9.6	8.4
									dSSI	179.0	184.0	172.9	189.8
**11**	M	64	160/100	34.3	A	3142.87	1.56	0.13	*SSI*	6.6	9.1	8.2	10.8
									dSSI	60.8	90.8	81.6	107.1
**12**	F	62	140/81	41.0	A	14164.13	2.12	0.08	*SSI*	37.9	33.7	30.0	44.6
									dSSI	1163.7	859.9	733.2	1443.1
**13**	M	80	163/100	48.2	A	2063.43	1.72	0.08	*SSI*	107.3	184.1	146.8	73.8
									dSSI	2000.9	3256.6	2589.8	1508.0
**14**	M	59	138/85	26.5	A	7170.91	1.73	0.09	*SSI*	10.3	9.9	10.3	10.2
									dSSI	176.3	185.3	193.3	167.5
**15**	M	65	140/88	31.0	A	4061.65	1.37	0.10	*SSI*	32.1	21.0	23.6	41.8
									dSSI	311.5	201.9	246.7	448.0

Case No.	Gender	Age	Pressure (mmHg)	Diameter (mm)	Group	λ_1	λ_2	λ_3	Index	A	P	L	R
**16**	M	69	141/87	27.3	A	12443.80	2.01	0.09	*SSI*	11.0	14.5	12.6	15.0
									dSSI	179.3	239.4	213.5	244.6
**17**	F	69	148/84	29.1	A	9984.55	1.97	0.09	*SSI*	10.7	13.2	11.3	21.5
									dSSI	203.0	232.4	199.7	311.3
**18**	M	73	160/100	33.5	A	4567.15	1.441	0.09	*SSI*	18.8	17.9	21.6	11.6
									dSSI	252.5	272.9	300.4	165.3
**19**	M	81	120/73	52.0	A	170699.31	2.41	0.05	*SSI*	76.7	83.2	72.0	85.7
									dSSI	1381.6	1569.8	1366.6	1546.1
**20**	M	73	143/95	38.9	A	10351.21	1.46	0.07	*SSI*	156.6	154.8	174.2	98.1
									dSSI	2272.5	3179.6	3506.7	1621.5
**21**	M	73	148/84	29.6	A	4763.58	1.35	0.09	*SSI*	42.8	34.3	57.0	34.0
									dSSI	432.1	339.8	552.8	301.6
**22**	M	62	138/85	28.9	A	6770.53	1.97	0.12	*SSI*	11.6	10.6	11.8	13.1
									dSSI	117.6	138.1	138.7	142.8
**23**	M	51	147/105	26.0	A	1275.83	1.43	0.12	*SSI*	5.6	5.3	4.2	4.4
									dSSI	71.5	68.9	64.8	64.2
**24**	M	69	113/87	46.6	A	33129.04	2.19	0.05	*SSI*	56.4	27.1	28.7	51.3
									dSSI	1454.8	801.0	823.0	936.8
**25**	M	70	154/103	27.0	A	1309.45	1.16	0.09	*SSI*	9.9	13.4	10.0	14.5
									dSSI	141.0	243.6	138.6	106.3
**26**	M	66	152/96	35.1	A	2147.92	1.48	0.15	*SSI*	20.2	15.9	15.0	15.7
									dSSI	106.2	230.0	80.5	116.5

Note:
"o" – Missing information
"H" – Healthy group
"A" – Aneurysm group

Table S3 Percentage difference in the tension when the assumed wall thickness is changed.

	Healthy Group			Aneurysm Group		
Case No.	1	2	3	11	12	13
Difference	2.25%	0.78%	0.51%	0.79%	1.02%	1.41%

Table S4 Material model sensitivity of tension analysis with inverse FEM

Material Model	iFEM setting	Lumen tension of Case 12	Error
Isotropic elastic	E=2e5 MPa<br>v=0.45	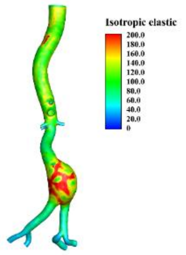	/
Neo-Hookean	E=2e5 MPa<br>v=0.45	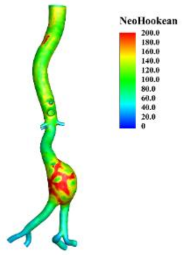	<0.01%
GOH	c=4e5 MPa<br>k1=2e5 MPa<br>k2=2e3<br>$\kappa = 0.226$<br>$\gamma = 49.98$<br>k=6e6 MPa	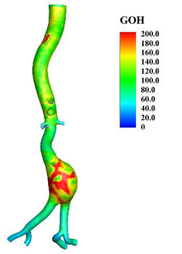	0.84%

Note: The error indicates the average difference of all the nodes on the model compared to the results of isotropic elastic model.
